# Mesenchymal stem cells with rhBMP-2 inhibits the growth of canine osteosarcoma cells

**DOI:** 10.1186/1746-6148-8-17

**Published:** 2012-02-22

**Authors:** Rose Eli Grassi Rici, Dayane Alcântara, Paula Fratini, Cristiane Valverde Wenceslau, Carlos Eduardo Ambrósio, Maria Angelica Miglino, Durvanei Augusto Maria

**Affiliations:** 1 Department of Surgery, Faculty of the Veterinary Medicine and Zootecny, São Paulo University, São Paulo, Brazil; 2 Laboratory of Animal Morphology, University Center of the Octavio Bastos Educational Foundation, São João da Boa Vista, Brazil; 3 Laboratory of Animal Anatomy - Faculty of Zootecny and Food Engineering, São Paulo University, São Paulo, Brazil; 4 Laboratory of Biochemical and Biophisic, Butantan Institute, São Paulo, São Paulo, Brazil; 5 Departamento de Cirurgia - Faculdade de Medicina Veterinária e Zootecnia, Universidade de São Paulo (USP), Av. Prof. Dr. Orlando Marques de Paiva, 87, Cidade Universitária, São Paulo, SP, CEP: 05508-270, Brazil

**Keywords:** Osteosarcoma, rhBMP-2, Mesenchymal stem cell, Canine

## Abstract

**Background:**

The bone morphogenetic proteins (BMPs) belong to a unique group of proteins that includes the growth factor TGF-β. BMPs play important roles in cell differentiation, cell proliferation, and inhibition of cell growth. They also participate in the maturation of several cell types, depending on the microenvironment and interactions with other regulatory factors. Depending on their concentration gradient, the BMPs can attract various types of cells and act as chemotactic, mitogenic, or differentiation agents. BMPs can interfere with cell proliferation and the formation of cartilage and bone. In addition, BMPs can induce the differentiation of mesenchymal progenitor cells into various cell types, including chondroblasts and osteoblasts. The aim of this study was to analyze the effects of treatment with rhBMP-2 on the proliferation of canine mesenchymal stem cells (cMSCs) and the tumor suppression properties of rhBMP-2 in canine osteocarcoma (OST) cells. Osteosarcoma cell lines were isolated from biopsies and excisions of animals with osteosarcoma and were characterized by the Laboratory of Biochemistry and Biophysics, Butantan Institute. The mesenchymal stem cells were derived from the bone marrow of canine fetuses (cMSCs) and belong to the University of São Paulo, College of Veterinary Medicine (FMVZ-USP) stem cell bank. After expansion, the cells were cultured in a 12-well Transwell system; cells were treated with bone marrow mesenchymal stem cells associated with rhBMP2. Expression of the intracytoplasmic and nuclear markers such as Caspase-3, Bax, Bad, Bcl-2, Ki-67, p53, Oct3/4, Nanog, Stro-1 were performed by flow citometry.

**Results:**

We evaluated the regenerative potential of *in vitro *treatment with rhBMP-2 and found that both osteogenic induction and tumor regression occur in stem cells from canine bone marrow. rhBMP-2 inhibits the proliferation capacity of OST cells by mechanisms of apoptosis and tumor suppression mediated by p53.

**Conclusion:**

We propose that rhBMP-2 has great therapeutic potential in bone marrow cells by serving as a tumor suppressor to increase p53 and the pro-apoptotic proteins Bad and Bax, as well as by increasing the activity of phosphorylated caspase 3.

**Study design:**

Canine bone marrow mesenchymal stem cells associated with rhBMP2 in canine osteosarcoma treatment: "*in vitro*" study

## Background

Osteosarcoma is as a primary bone cancer common in dogs. Frequently, osteosarcoma affects the limb bones of large-sized dogs over 15 kg at an average age of 7 years [1]. In 75% of cases, osteosarcoma affects either the appendicular skeleton [2] or the pelvic and thoracic limbs, and in the remaining 25%, it affects the axial skeleton or the flat bones [3,4]. Generally, males have a higher incidence of osteocarcoma than females [2], with the exception of the St. Bernard, Rottweiler, and Danish breeds, in which females are most affected [5,6]. Osteosarcoma cells induce platelet aggregation, which facilitates metastasis formation. Platelet aggregation and metastasis most commonly occur in the lung [7]. Platelet aggregation promotes the establishment of tumor cell aggregates, which could serve as a bridge between the tumor cells and the vascular surfaces [6].

A primary extraskeletal osteosarcoma has a metastatic rate that ranges from 60 to 85% in dogs and an average life expectancy after surgery of 26-90 days, which varies according to the location where the metastasis occurs [4].

Metastasis is the most common cause of death in dogs with osteosarcoma, and 90% of dogs either die or are euthanized due to complications associated with lung metastases. Therefore, chemotherapy is used to increase the long-term survival of dogs with osteosarcoma. To reduce the occurrence of metastasis, chemotherapy is often used in combination with surgery or radiotherapy. Specifically, either cisplatin or cisplatin and doxorubicin are chemotherapeutic agents used in dogs [8,9]. Numerous studies have aimed to develop antiangiogenic therapeutic strategies, which can be combined with other treatments [10].

The bone morphogenetic proteins (BMPs) belong to a unique group of proteins that includes the growth factor TGF-β. BMPs play important roles in cell differentiation, cell proliferation, and inhibition of cell growth. They also participate in the maturation of several cell types, depending on the microenvironment and interactions with other regulatory factors [11,12]. Depending on their concentration gradient, the BMPs can attract various types of cells [13] and act as chemotactic, mitogenic, or differentiation agents [14]. BMPs can interfere with the proliferation of cells and the formation of cartilage and bone. Finally, BMPs can also induce the differentiation of mesenchymal progenitor cells into various cell types, including chondroblasts and osteoblasts [15].

BMPs play important roles in cell differentiation, proliferation, morphogenesis, and apoptosis, and recent studies have shown that recombinant human BMP-2 (rhBMP-2) inhibits tumor formation [16-19]. However, the role of rhBMP-2 in canine osteosarcoma remains unknown. The osteoinductive capacity of rhBMP-2 has been widely studied in preclinical models and evaluated in the clinical setting [20]. Gene and cell therapy studies have shown that many bone defects can be treated by implantation of resorbable polymers with bone marrow cells transduced with an adenovirus expressing rhBMP-2 [21]. In addition, rhBMP-2 can be used as a substitute for bone grafts in spinal surgery, with results comparable to autogenous grafts [22].

Based on the studies cited above, the present work explores the proliferative effects of canine mesenchymal stem cells (cMSCs) and osteosarcoma (OST) cells treated with rhBMP-2 to evaluate their regenerative potential in the presence of the *in vitro *treatment.

## Methods

### Isolation of canine osteosarcoma (OST) cells

The osteosarcoma cell lines were isolated from biopsies and excisions of animals with osteosarcoma performed in veterinary clinics and hospitals in São Paulo and were characterized by the Laboratory of Biochemistry and Biophysics, Butantan Institute. Approved by Ethic Committee in the use of animals, protocol number 1654/2009. After harvesting the tissue, the fragments were washed with saline solution containing antibiotics (10%), and the fatty and hemorrhagic tissues were removed from the tumors. Next, the tumors were divided in two samples. The first sample was cut into pieces of 0.01 cm^2 ^using a scalpel and adhered to Petri dishes for 1 h in fetal bovine serum (FBS) in an incubator at 37°C and 5% CO_2_. The second sample was used for histopathological diagnosis. The cells were cultured in 25-cm^2 ^flasks with DMEM-H (LGC) media supplemented with 10% of FBS (VITROCELL), 1% of penicillin and streptomycin (GIBCO), and 1% of pyruvic acid (GIBCO) at pH 7.4 and were kept in a incubator at 37°C and 5% CO_2_. The cells grew in a monolayer attached to the surface of the culture plate. The sub-culture of cells was performed by trypsinizaton of the confluent monolayer cell cultures every 3 days.

### Isolation of mesenchymal stem cells derived from canines fetuses (cMSCs)

The mesenchymal stem cells were derived from the bone marrow of canine fetuses (cMSCs) and belong to the University of São Paulo, College of Veterinary Medicine (FMVZ-USP) stem cell bank. Approved by Ethic Committee in the use of animals, protocol number 931/2006. cMSCs were cultured in 25 cm^2 ^flasks with ALPHA-MEM media (VITROCELL) supplemented with 10% of HyClone FBS, 1% of penicillin and streptomycin (GIBCO), 1% of nonessential amino acids (GIBCO), and 1% L-glutamine (GIBCO) at pH 7.4, and they were kept in an incubator at 37°C and 5% CO_2_. The cells grew in a monolayer attached to the surface of the culture plate. The sub-culture of cells was performed from the monolayer cells.

### *In vitro *treatment of osteosarcoma with stem cells derived from canine bone marrow and rhBMP-2

After expansion, the OST and cMSCs cells were cultured in a 12-well plates containing 10^5 ^cells per well. The groups was composed by OST cells untreated (control group 1) and cMSCs untreated (control group 2).

These cells were treated with different concentrations of rhBMP-2 (5, 10, and 20 nM) for 120 h. Cells were labeled with carboxyfluorescein diacetate succinimidyl diester (CFSE; Invitrogen, Carlsbad, CA, USA) by incubation for 15 min at room temperature with 1 mM CFSE at a density of 2 × 10 ^6 ^cells/ml in PBS and the reaction was stopped by adding bovine serum to the PBS. At different times of culture, the cells were harvested and directly run on the FACSCalibur flow cytometer (Becton Dickinson) to measure the intensity of CFSE in the cells in order to monitor the number of times that the cells had divided during culture.

Then a total of 3 × 10^5 ^cells were cultured in 96 U-bottom well plates and were kept in an incubator at 37°C and 5% CO_2 _for 5 days. After the incubation period, the proliferation of the OST cells and the cMSCs was measured to standardize the optimal concentration to be used in the *in vitro *regenerative therapy of canine osteosarcoma.

For transwell culture the cMSCs treated with rhBMP2 (20 nM) were cultured onto dry 6.5 mm diameter, 0.4 μm pore size polycarbonate Transwell filters containing 10^5 ^cells per filter. OST cells treated with rhBMP2 (5 nM) was added to the lower well, containing 10^5 ^cells per well. This treatment lasted for 120 h.

### Expression of cell proliferation and cell death markers

The cells obtained from the Transwell culture plates were trypsinized and inactivated with FBS, centrifuged at 1,500 rpm for 10 min, and the supernatant was discarded. The pellet was resuspended in 5 ml of PBS at a concentration of 10^6 ^cells/ml. To analyze the amounts of intracytoplasmic and nuclear markers, the cells were permeabilized with 5 μl of 0.1% Triton X-100 for 30 min before the addition of specific primary antibodies. The following markers were used to determine the cell death pathways: caspase-3, Bax, Bad, and Bcl-2. The antibodies for Ki-67 and p53 were used to determine the proliferation index. Mesenchymal stem cell differentiation and maturation was determined by using the Oct 3/4, Stro-1, and Nanog antibodies. The samples were analyzed in a flow cytometer (FACSCalibur), and the expression of the markers was determined by comparison with an isotype control.

### Statistical analysis

All the values reported are mean ± SD. Statistical analyses were performed using GraphPad Prism Version 5 software and significance was determined using either the nonparametric MannWhitney test for unpaired data or the two-tailed *t*-test. Difference was considered significant at *p *< 0.05. In all graphs, *, **, *** indicate difference between groups at *p *< 0.05, 0.01 and 0.001, respectively.

## Results

### Proliferative effects of rhBMP-2 in canine mesenchymal stem cells (cMSCs) and canine osteosarcoma (OST) cells

**Figure 1:**
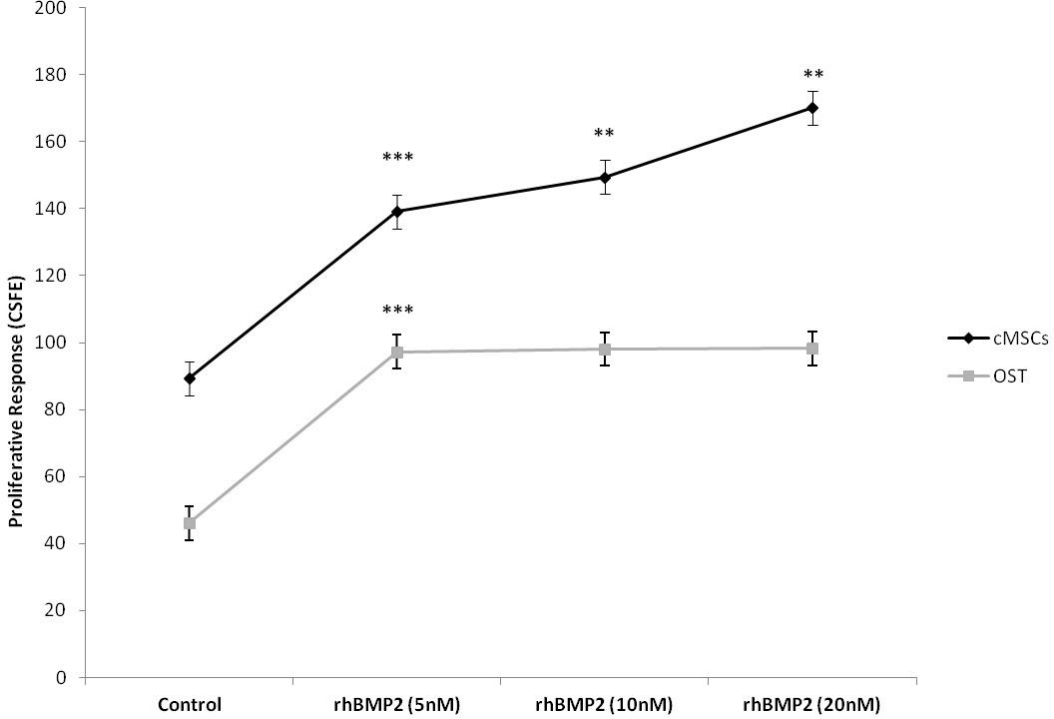
**Proliferation curves**. Proliferation curves of bone marrow stem cells derived from canine fetuses (cMSCs) and canine osteosarcoma (OST) cells 120 h after rhBMP-2 treatment. The cells were stained with CSFE and analyzed by flow cytometry. Statistical differences were obtained by analysis of variance (*** *p *< 0.001, ** *p *< 0.01, * *p *< 0.05).

cMSCs cells showed an enhanced cellular proliferation response in comparison to OST cells when treated with 20 nM of rhBMP-2 for 120 h. A significant proliferative response was observed in OST after treatment with 5 nM of rhBMP-2 (Figure 1).

### Expression of cellular markers in canine osteosarcoma cells treated with cMSCs and rhBMP-2

**Figure 2:**
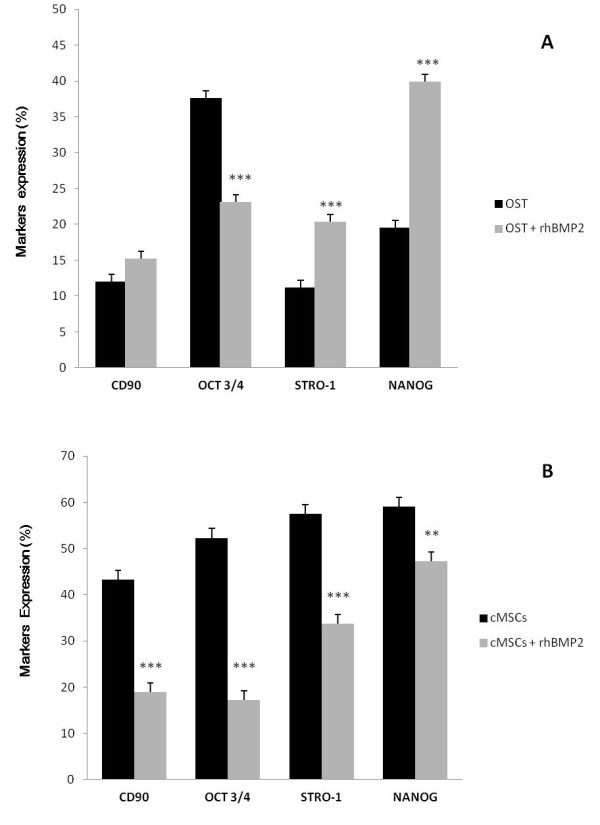
**Pluripotent embryonic stem cell markers**. Expression of pluripotent embryonic stem cell markers (Nanog and Oct 3/4) and markers of mesenchymal stem cells with osteogenic potential (Stro-1 and CD90) in OST cells (**A**) and cMSCs (**B**) from the control and the group treated with rhBMP-2 (5 nM e 20 nM respectively) for 120 h. Statistical differences were obtained by analysis of variance (*** *p *< 0.001, ** *p *< 0.01).

Oct 3/4 and Nanog are markers of pluripotent embryonic stem cells. We analyzed the expression of these markers in cMSCs treated with rhBMP-2 (20 nM) decreased the expression of Oct 3/4 and Nanog in the bone marrow cells. Stro-1 and CD90 are surface markers of mesenchymal stem cells that specifically possess osteogenic potential. We observed an increase in the Stro-1 marker when the osteosarcoma cells were treated with rhBMP-2 (5 nM). This increase correlates with the finding that rhBMP-2 is expressed in some types of osteosarcoma, as observed in the OST cells (Figure 2A). A significant reduction in the expression of the pluripotent embryonic stem cell markers (Oct 3/4 and Nanog) and the mesenchymal stem cells markers (CD90 and Stro-1) was observed in the canine bone marrow cells treated with rhBMP-2, as shown in Figure 2B.

### Flow cytometric analyses of markers of proliferation and cell death by apoptosis or necrosis

**Figure 3:**
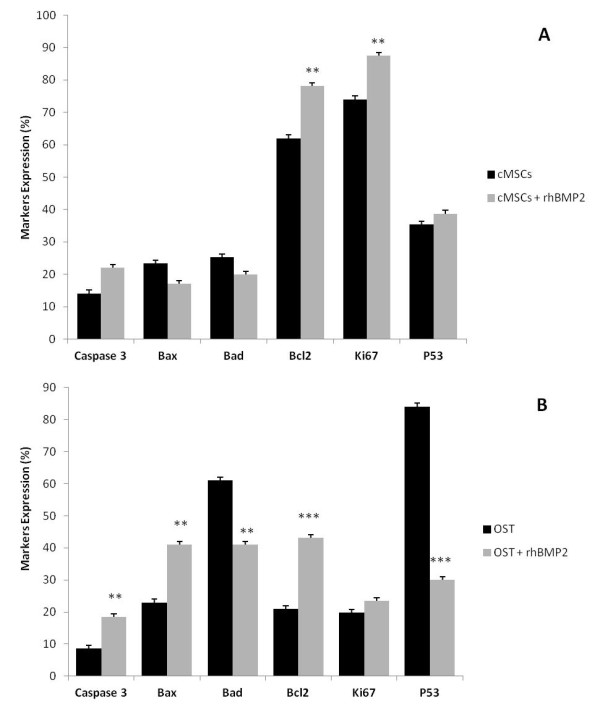
**Pro and anti-apoptotic proteins and the proliferative capacity**. Expression of pro- and anti-apoptotic proteins and the proliferative capacity in cMSCs (**A**) and OST cells (**B**) 120 h after treatment with rhBMP-2 (20 nM and 5 nM respectively) analyzed by flow cytometry. Statistical differences were obtained by analysis of variance (*** *p *< 0.001, ** *p *< 0.01).

The treatment of cMSCs with rhBMP-2 induced an increase in proliferation followed by a significant increase in the expression of the Ki-67 marker. We observed a decrease in the expression of p53, which is involved in the regulation of apoptosis and tumor suppression. Treatment with rhBMP-2 can activate p53 when DNA damage is present, and this occurs without the involvement of cMSCs in the tumorigenic processes or an increase in apoptosis via phosphorylated caspase-3. We also observed a significant increase in the expression of the anti-apoptotic protein Bcl-2 and a decrease in phosphorylated caspase-3 after treatment with rhBMP-2. There was a inhibition of cell proliferation in the OST cells treated with rhBMP-2, which promotes a significant increase in p53 expression. An increased induction of cell death mediated by pro-apoptotic proteins (Bax) was observed, resulting in suppression of proliferation and an increase of phosphorylated caspase-3 (Figure 3).

**Figure 4:**
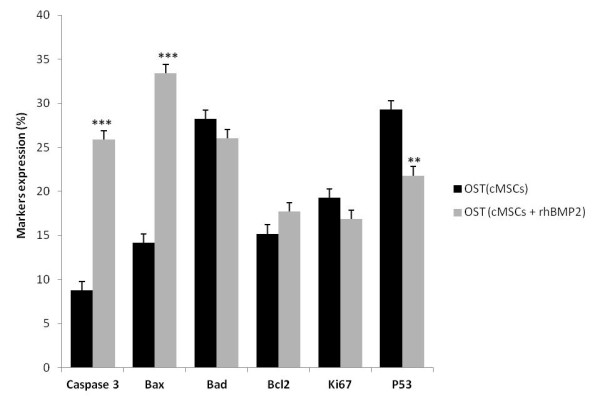
**Pro apoptotic effects of osteosarcoma treatment with cMSCs associated with rhBMP2**. Markers expression of osteosarcoma treatment with cMSCs and cMSCs treated with rhBMP2 (5 nM) for 120 after treatment analyzed by flow cytometry. Statistical differences were obtained by analysis of variance (*** *p *< 0.001, ** *p *< 0.01).

**Figure 5:**
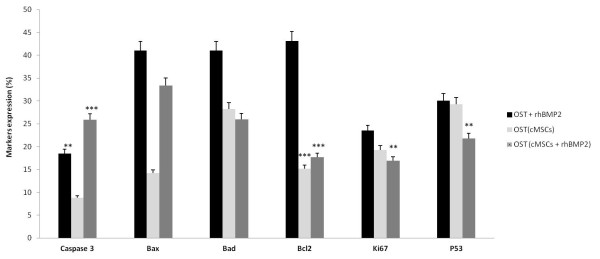
**Comparison between different treatments**. Markers expression of osteosarcoma treatment with cMSCs, rhBMP2 and cMSCs treated with rhBMP2 (5 nM) 120 h after treatment analyzed by flow cytometry. Statistical differences were obtained by analysis of variance (*** *p *< 0.001, ** *p *< 0.01).

The treatment of osteosarcoma with cMSCs treated with rhBMP-2 induced a significant decrease in the expression of the P53 marker which is involved in the regulation of apoptosis and tumor suppression. We observed a decrease in the expression of Ki67, which is involved in the cellular proliferation. The treatment had shown a significant increase in apoptosis via phosphorylated caspase-3 and in the expression of the pro-apoptotic protein Bax and a significant decrease in the expression of the anti-apoptotic protein Bcl-2 (Figures 4 and 5).

## Discussion

In the present study, we analyzed the effects of rhBMP-2 in mesenchymal stem cells for use in the regenerative therapy of canine osteosarcoma utilizing OST cells as a model. The OST cells remained spindle-shaped during cell growth and the confluent stages.

Treatment of OST cells with rhBMP-2 compromised the osteoblastic phenotype. Osteoprogenitors are either pre-determined or inducible, depending on additional signals necessary to cause differentiation. This difference is important because it reflects the variation between cell compromise, which is when cell fate is programmed, and cell differentiation, which is when cells are compromised by permissive microenvironment signs [23].

Although the exact function and interrelation of each type of BMP are not completely understood, evidence indicates that they are a part of a series of complex factors that regulate cell differentiation, specifically maturation intochondroblasts and osteoblasts. The structural and functional evolutionary conservation of genes encoding BMPs suggest that these genes have critical regulatory functions in the process of differentiation during development and neoplastic transformation. In human colorectal carcinoma, for example, rhBMP-2 acts as a tumor suppressor [24]. Similarly, rhBMP-2 shows anti-proliferative and pro-apoptotic potential in gastric, prostate, and ovarian cancer cells. In breast cancer cell lines, rhBMP-2 treatment decreases cell proliferation. The effects of the BMPs varies with tumor progression. In other words, in the early stages of carcinogenesis, the TGF beta superfamily acts by suppressing tumor growth, and at later stages, the superfamily actually promotes tumor progression [25].

In our findings, we observed that when mesenchymal stem cells were exposed to rhBMP-2, there was a significant reduction in the expression of the marker Nanog. We noted a significant decrease in expression of the cell proliferation marker Oct 3/4 in OST cells treated with rhBMP-2. The treatment of OST cells with rhBMP-2 after Transwell culture inhibited the proliferative response (Ki-67 expression) and promoted an increase in cell death mediated by the pro-apoptotic proteins (Bax and Bad), resulting in suppression of proliferation and an increase of phosphorylation of caspase-3.

The treatment of bone marrow cells with rhBMP-2 stimulates the production of growth and differentiation factors. Thus, rhBMP-2 treatment may be a relevant treatment for canine osteosarcoma cells. Eliseev et al. [26], showed that rhBMP-2 increases the expression of Bax via Runx2, thus increasing the sensitivity of osteosarcoma cells to apoptosis. In our experiments, we also observed an increase in Bax expression when canine osteosarcoma cells were treated with rhBMP-2. These results suggest that rhBMP-2 treatment increases the susceptibility of OST cells to death by apoptosis.

Kawamura et al. [17] observed that rhBMP-2 inhibits the growth of human multiple myeloma U266 cells by arresting the cells in the G1 phase of the cell cycle, leading to apoptosis. The combined treatment with rhBMP-2 induces cell cycle inhibitory proteins, such as p21 and p27, and induces other proteins associated with apoptosis, such as Bcl-xl, Bcl2, Bax, and Bad. Thus, rhBMP-2 may be an important tool for the treatment of multiple myeloma due to its anti-tumor and bone regeneration effects. Kawamura et al. [17] investigated the antiproliferative effect of rhBMP-2 in myeloma cells and found that BMPs inactivate the STAT3 protein, which is a signal transducer activated by IL-6. BMPs were also found to increase the expression of cell cycle inhibitors leading to a cell replication blockage via pRb.

Based on the studies of Hsu et al. [27], BMPs can function either as an oncogene or as a tumor suppressor, depending on the stage of disease. The effects of BMPs are cell type-specific and may vary among different tumors. BMPs are also reported as tumor suppressors and act on the cell cycle by inducing apoptosis of abnormal cells, such as tumors. Hardwick et al. [24] used cell lines of colorectal cancer to evaluate the role of rhBMP-2. They observed that rhBMP-2 reduced cell growth and stimulated apoptosis due to high levels of phosphorylated caspase-3. In our results, we also observed a decrease in cell growth when OST cells were treated with rhBMP-2, and we observed an increase of caspase-3 levels.

Treatment with rhBMP-2 may be a new therapeutic option for canine osteosarcoma. We found that rhBMP-2 decreases the expression of embryonic stem cell markers Nanog and Oct 3/4 in different treatment regimens, and it is also associated with tumorigenesis of many types of cancer [28,29]. Because rhBMP-2 has the potential to inhibit the expression of markers such as Nanog and Oct 3/4, it may also exhibit anti-tumor effects in animal models *in vivo*. Oct 3/4 and Nanog are important factors in the regulation of self-renewal and the pluripotency of embryonic stem cells. There are studies showing a correlation of these cells with cases of tumorigenesis [29]. Oct 3/4 is a marker for both adult stem cells and cancer stem cells. Inhibition of this factor can inhibit the expression of proteins associated with tumorigenesis.

Osteogenesis is defined by a series of events, which starts with a commitment to an osteogenic lineage by mesenchymal cells. Subsequently, these cells proliferate and demonstrate an upregulation of osteoblast-specific genes and mineralization. Multiple signaling pathways have been demonstrated to participate in the differentiation of an osteoblast progenitor to a committed osteoblast [30,31].

An association between the expression of STRO-1 and the presence of cells with osteogenic potential has been demonstrated in precursor adult human bone marrow. STRO-1^+ ^population of human bone marrow cells is capable of osteogenic differentiation. The expression STRO-1 is complicated by the fact that a considerable proportion of STRO-1^+ ^cells are not of the osteogenic lineage and the exact stage of osteogenic differentiation at which STRO-1 is expressed remains unclear, especially when working with cultured cell populations and the coexpression of STRO-1 and a panel of antibodies recognizing cell surface determinants which may be regulated during osteogenic differentiation [32].

BMP-2 alone does not induce osteogenesis in isolates of human bone marrow stromal cells as measured by stimulation of alkaline phosphatase expression. However, BMP-2 does induce other markers associated with differentiation of osteoblasts. This osteogenic capacity is seen in stromal cells isolated from mice, rats, rabbits, and humans; however, cell behavior and efficacy of inducers varies in a species-dependent manner [33].

BMP-2 stimulates surrounding tissues; however, more robust data is needed to demonstrate that BMP-2 also augments the osteogenic potential of implanted MSCs cells. In the present study, probably the effects of MSCs and rBMP-2 treated model culture Transwell, controlled environmental niches and alterations in this microenvironment can dramatically modify their behavior and differentiation capacities.

Langenfeld et al. [34] showed that cell culture conditions and the intra- and extra-cellular antagonist concentrations interfere with the biological activities of BMPs. Wang et al. [23] observed that rhBMP-2 inhibits the tumorigenic potential of human osteosarcoma OS99-1 cells. The inhibition was due to a decrease in the expression of proteins associated with tumorigenesis and an increase of osteosarcoma cell differentiation in response to rhBMP-2. Thus, rhBMP-2 could be considered a novel tool for the treatment of human osteosarcoma. Our study clearly showed that the association of mesenchymal stem cells derived from canine fetal bone marrow, combined with rhBMP-2, decreases the tumorigenic potential of canine osteosarcoma cells *in vitro*.

## Conclusions

The *in vitro *treatment of bone marrow cells with rhBMP-2 decreased their osteogenic potential. Thus, we suggest that the treatment conditions for both osteogenic induction and for tumor regression are favorable when associated with stem cells derived from canine bone marrow. cMSCs treated with rhBMP-2 inhibits the proliferation capacity of OST cells by mechanisms of apoptosis and tumor suppression mediated by p53. Treatment of bone marrow cells with rhBMP-2 showed a high therapeutic potential observed by the increase in the tumor suppressor protein p53 and pro-apoptotic proteins Bad and Bax, and by increased activity of phosphorylated caspase-3.

## Competing interests

The authors declare that they have no competing interests.

## Authors' contributions

REGR, DA, CVW collected the materials, established cell lines and carried out the experiment. REGR, DAM and PF performed the cytometry analysis and wrote manuscript. CEA and MAM reviewed the manuscript and the quality of the written English. All authors read and approved the final paper.
